# Supplementary material for: Yiai Fuzheng decoction inhibits triple-negative breast cancer by remodeling the immune microenvironment
Source: Front Immunol. 2025 Sep 30;16:1615631. doi: 10.3389/fimmu.2025.1615631 (PMC12518410; doi:10.3389/fimmu.2025.1615631)
Supplement: Supplementary file 2 [file DataSheet1.zip › Supplementary File 2/Treatment/D_vs_H/07_Pathway_Analysis/Pathway_Analysis/Sig_Pathways_Name/Citrate cycle (TCA cycle).pdf]

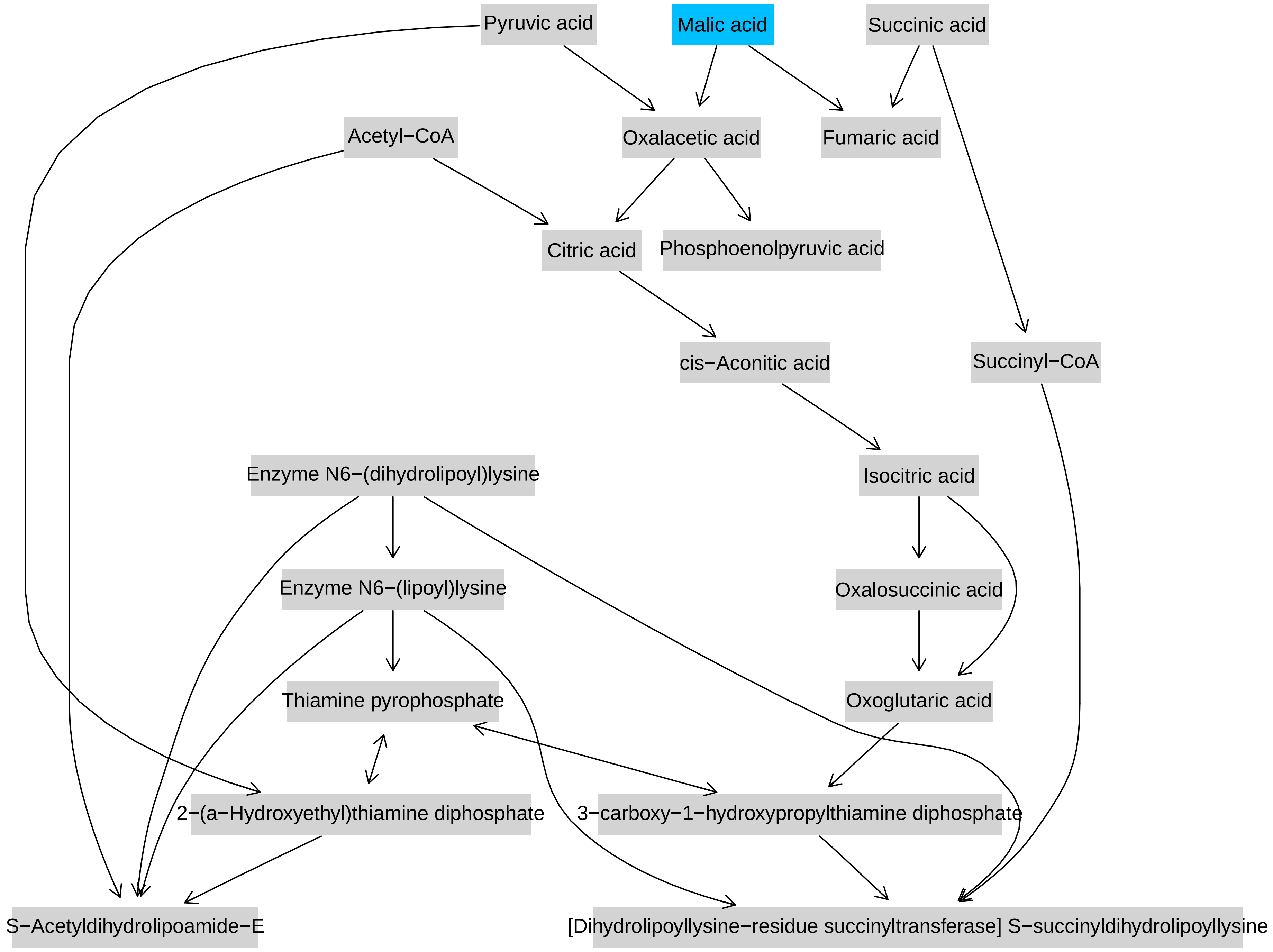
Pyruvic acid
Malic acid
Succinic acid
Acetyl−CoA
Oxalacetic acid
Fumaric acid
Citric acid
Phosphoenolpyruvic acid
cis−Aconitic acid
Succinyl−CoA
Enzyme N6−(dihydrolipoyl)lysine
Isocitric acid
Enzyme N6−(lipoyl)lysine
Oxalosuccinic acid
Thiamine pyrophosphate
Oxoglutaric acid
2−(a−Hydroxyethyl)thiamine diphosphate
3−carboxy−1−hydroxypropylthiamine diphosphate
S−Acetyldihydrolipoamide−E
[Dihydrolipoyllysine−residue succinyltransferase] S−succinyldihydrolipoyllysine
